# Peripheral Pathways in the Food-Intake Control towards the Adipose-Intestinal Missing Link

**DOI:** 10.1155/2013/598203

**Published:** 2013-12-05

**Authors:** Hugo Mendieta Zerón, Ma. Victoria Domínguez García, María del Socorro Camarillo Romero, Miriam V. Flores-Merino

**Affiliations:** ^1^Medical Sciences Research Center (CICMED), Autonomous University of the State of Mexico (UAEMex), 50170 Toluca, Mexico; ^2^Asociación Científica Latina (ASCILA) and Ciprés Grupo Médico (CGM), Felipe Villanueva sur 1209 Col. Rancho Dolores Z.C., 50170 Toluca, Mexico

## Abstract

In the physiological state a multitude of gut hormones are released into the circulation at the same time depending on the quality and quantity of the diet. These hormones interact with receptors at various points in the “gut-brain axis” to affect short-term and intermediate-term feelings of hunger and satiety. The combined effects of macronutrients on the predominant gut hormone secretion are still poorly understood. Besides, adipokines form an important part of an “adipoinsular axis” dysregulation which may contribute to **β**-cell failure and hence to type 2 diabetes mellitus (T2DM). Even more, gestational diabetes mellitus (GDM) and T2DM seem to share a genetic basis. In susceptible individuals, chronic exaggerated stimulation of the proximal gut with fat and carbohydrates may induce overproduction of an unknown factor that causes impairment of incretin production and/or action, leading to insufficient or untimely production of insulin, so that glucose intolerance develops. The bypass of the duodenum and jejunum might avoid a putative hormone overproduction in the proximal foregut in diabetic patients that might counteract the action of insulin, while the early presentation of undigested or incompletely digested food to the ileum may anticipate the production of hormones such as GLP1, further improving insulin action.

## 1. Introduction

Under steady-state conditions, all ingested fuels (energy intake) are normally metabolized to maintain basic metabolic rate, thermogenesis, and muscle action (energy expenditure). Food intake and energy expenditure can be influenced by environment and lifestyle. This knowledge highlights the importance of understanding the physiological and molecular mechanisms responsible for the final predominated signal of appetite control [1].

**Figure 1:**
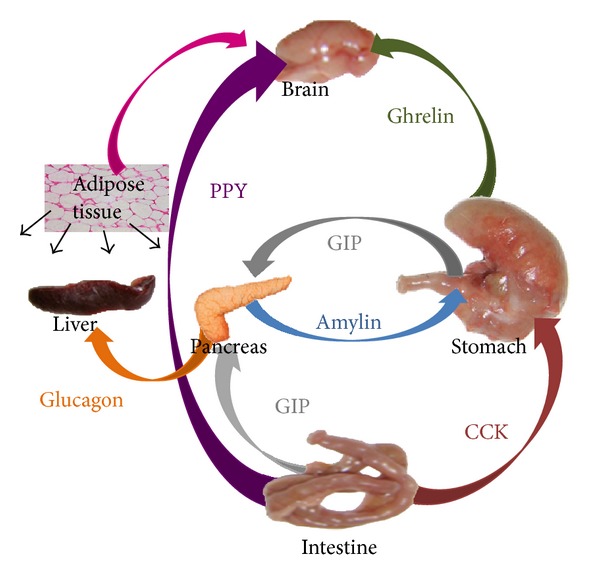
Interacting hormones involved in food-intake control. GIP: glucose-dependent insulinotropic polypeptide; PYY: peptide YY.

All of the peripheral and central processes that make up this highly complex system are subject to individual predisposition through genes. Key peripheral components are the gustatory system, the gastrointestinal tract, pancreas, liver, muscle, and adipose tissue (Figure 1). The aim of this review is to give an insight into the major peripheral signals in the food intake control, viewed in a dynamic context taking into account the major food related signals and to discern possible explanations of the diabetogenic state recovery after weight lose [2].

## 2. Intestinal Signals

Over 30 different regulatory peptide hormones are secreted in the gut, the largest endocrine organ in the body. Gut nutrient content stimulates several of these hormones which interact with receptors at various points in the “gut-brain axis” to affect short-term and intermediate-term feelings of hunger and satiety [3]. The major gut hormones implicated in appetite control (Table 1) are age, sex, and body mass index (BMI) dependent (Table 2).

By chemo/mechanosensory mechanisms, the gastrointestinal tract sends information to the brain regarding available energy for metabolism. Postprandially, activation of gut mechanoreceptors, changes in circulating nutrient concentration, and release of anorectic gut hormones all lead to a reduction in subsequent feeding [4]. However, apart from traditional homeostatic feedback regulation of energy balance, food appearance, flavor, and availability in addition to social, cultural, and economic influences determine food intake. Importantly, there is also modulation of food intake by hedonic and mnemonic neuronal circuits [5]. The modern consensus is, therefore, that there is interaction between homeostatic and nonhomeostatic inputs, which together lead to coordination in terms of inducing either an orexigenic or anorectic response. 

### 2.1. Amylin

Amylin or islet amyloid polypeptide (IAPP) is a 37-amino acid pancreatic peptide that is cosecreted with insulin. This hormone is a member of the calcitonin family of peptides and is involved in meal satiety signaling [6–8]. As such, amylin and related compounds also inhibit gastric emptying and reduce meal size [9, 10].

Synthetic or naturally occurring amylin agonists have been shown to be more potent and have a longer duration of feeding suppression than amylin itself [11]; one such potent anorectic analog in humans, primates, and rodents is calcitonin of salmon origin (sCT) [12]. This compound irreversibly binds to amylin receptors to produce sustained anorectic responses [13]. Additionally, the anorectic potency of amylin agonists is not dependent on intact vagal afferent signaling [14].

### 2.2. Cholecystokinin

Cholecystokinin (CCK) is considered a highly selective satiation signal acting over two receptors, the CCK-B, predominantly found in the brain, where the unsulfated tetrapetide CCK-4 is active and the A-type receptor of the gastrointestinal tract, where the sulfated CCK-forms (CCK-8-S, CCK-33-S, CCK-39-S, and CCK-58-S) bind. Satiety and meal size limitation are mediated mainly by the CCK-A-receptor [27].

Luminal fat and protein are strong releasers of CCK from enteroendocrine cells. The food intake suppressive effect results from CCK acting in a paracrine fashion on CCK-A receptors located on vagal sensory nerve terminals in the mucosal lamina propia [28–30]. Intact vagal afferent neurons are required for the satiety effects of CCK.

### 2.3. Endocannabinoid System

There are reports suggesting that the peripheral endocannabinoid system is implicated in the regulation of energy balance. For instance, during periods of fasting, levels of the endocannabinoid and anandamide are elevated in the rat duodenum [31]. Furthermore, in obese rodents, an increase in mRNA for CB1 receptors is observed in the stomach [32] and in the nodose ganglia [33], and endocannabinoid levels in the duodenum, pancreas, and liver are similarly elevated in this animal model [34]. On the contrary, cannabinoid CB1 receptor antagonists reduce food intake and body weight, but clinical use in humans has been limited by effects on the central nervous system (CNS), although there are new options with limited CNS penetration [35]. 

### 2.4. Ghrelin

Ghrelin is an orexigenic hormone [36], secreted in the oxyntic gland cells in the mucosa of the stomach, originally isolated from the rat stomach as an endogenous ligand of the growth hormone secretagogue receptor (GHS-R), and has been shown to have a GH-releasing effect [37]. Yet, the ghrelin receptor is expressed by a subset of stomach innervating vagal afferent neurons in the nodose ganglia [38]. 

Plasma ghrelin concentrations are elevated during a fast. Moreover, plasma ghrelin concentrations display a circadian rhythm: a rise before each meal and a rapid fall after eating. Fasting morning ghrelin concentrations have a negative correlation with fat mass index [39]. On the other hand, diet-induced weight loss in obese individuals increased plasma ghrelin levels [40]. These findings suggest that plasma ghrelin levels may represent a compensatory response to altered energy metabolism. Of note, central and peripheral administration of ghrelin stimulates food intake and body weight gain [37]. 

### 2.5. Glucose-Dependent Insulinotropic Polypeptide (GIP)

Glucose-dependent insulinotropic polypeptide (GIP) is manufactured and released in the duodenum and proximal jejunum by the K cells. Its plasma concentration increases quickly following food ingestion, stimulating an increase in insulin synthesis and secretion [41]. Carbohydrate, fat, and protein have all been shown to stimulate GIP secretion [42]. Pancreatic and duodenal homeobox-1 (Pdx-1) binds to GIP promoter. This, together with the fact of a remarkable reduction in the number of GIP-expressing cells in Pdx−/− mice [43], suggests that this incretin may play a role in *β*-cell differentiation.

### 2.6. Glucagon-Like Peptide-1 (GLP-1)

Proglucagon is a 160-amino acid prohormone that is produced in the *α* cells of the pancreatic islets, the L cells of the distal gut, and within the CNS. Selective posttranslational proteolysis of proglucagon by prohormone convertases 1 and 2 results in the tissue-specific production of a number of biologically active fragments.

GLP-1 is a potent insulin secretagogue that is secreted, in response to ingested nutrients. GLP-1 and related agonists, such as exendin-4, have been demonstrated to reduce food intake by slowing gastric emptying, reducing meal size, and promoting feelings of satiety [44, 45]. The reductions in food intake by these compounds appear to be peripherally mediated, as they are dependent on intact vagal afferent signaling [46]. The importance of the vagus nerve in mediating the proximal-distal loop was elucidated from the evidence that GLP-1 secretion is enhanced when the fat is administered into the duodenum or when the GLP-1 secretion, in response to the infusion of physiological concentration of GIP, was completely abrogated by vagotomy [47].

### 2.7. Oxyntomodulin

Another product of the tissue-specific differential cleavage of proglucagon is oxyntomodulin (OXM), a hormone cosecreted with GLP-1 and PYY_3-36_ into the circulation by intestinal L-cells after nutrient ingestion [48]. OXM is a satiety signal through GLP-1R [36, 49] and administration reduces energy intake in both rodents and humans [50, 51]. OXM levels are increased after gastric bypass surgery.

### 2.8. PYY

PYY is a 36-amino acid peptide, which belongs to the pancreatic polypeptide (PP) family, which also includes NPY. All these bind to G-protein coupled receptors Y_1_, Y_2_, Y_4_, Y_5_, and Y_6_, displaying promiscuity in their interactions with these receptors by virtue of their shared hair-pin-fold motif structure [3].

PYY is produced by the L cells of the gut, with highest concentrations found in the large bowel and the rectum [52]. Two endogenous forms, PYY_1-36_ and PYY_3-36_, are released postprandially into the circulation. PYY_3-36_, which acts mainly via the Y_2_ receptor, is further produced by cleavage of the Tyr-Pro amino terminal residues of PYY_1-36_ by the enzyme dipeptidyl peptidase IV (DPP-IV). PYY_1-36_ predominates in the circulation in the fasted state, whereas PYY_3-36_ is the major circulating form postprandially. Following a meal, circulating levels of PYY_3-36_ rise within 15 min, peak at approximately 90 min and remain elevated for up to 6 hours [53]. The magnitude of the rise in PYY_3-36_ is in proportion to the calories ingested [54]. When exogenously administered intravenously, its circulating half-life is approximately 8 min [43].

Initial postprandial release of PYY_3-36_ is likely to be under neural control, and further release of PYY_3-36_ is observed when the nutrients arrive in the distal gut, particularly stimulated by a high fat diet [55]. The protein content of the diet is thought to be influential for delayed PYY_3-36_ release approximately 2 hours postprandially [56]. Besides a direct central action, PYY_3-36_ is likely to affect appetite via its effects on gut motility, leading to a sensation of fullness and satiety [57].

## 3. Adipose Signals

Adipokines form an important part of an “adipoinsular axis,” dysregulation of which may contribute to *β*-cell failure and hence to T2DM. While some adipokines have beneficial effects, others have detrimental properties depending on the predominant intracellular signalling pathways [58]. The major cause of T2DM could be a human metabolic zwitterion-like molecule, with positive or negative effects over the beta cell depending on its state of activation.

### 3.1. Adiponectin

Unlike many other adipokines, adiponectin has beneficial effects improving insulin sensitivity and vascular function, thus being both antidiabetic [59] and antiatherogenic [60]. Opposite to other adipokines the circulating levels are decreased when the BMI is higher. The loss in body weight by adiponectin is mainly due to stimulation of energy expenditure [61].

Two adiponectin receptors AdipoR1 and AdipoR2 that exhibit 67% homology have been cloned. Many of the effects of the adiponectin-AdipoR interaction have been suggested to be mediated by 5′ AMP-activated protein kinase (AMPK), peroxisome proliferator-activated receptor a (PPARa), and p38 mitogen-activated protein kinases (MAPK) [62].

### 3.2. Leptin

Leptin is thought to signal longer-term energy status. This hormone engages a number of intracellular pathways, including those associated with cyclic adenosine monophosphate (cAMP), MAPK, phosphatidylinositol 3′-kinase (PI3 K), and signal transducer and activator of transcription 3 (STAT3) [63, 64]. 

In contrast to adiponectin, serum concentration of circulating leptin is elevated in obesity. Thus, probably there is a decrease in response to leptin [61]. Although leptin showed a great potential in preclinical studies, it was usefulness in clinical trials [65]. In eating disorders the results are controversial [66].

In the hypothalamic arcuate nucleus there are two types of neuronal populations with high levels of expression of the leptin receptor (LEPR), proopiomelanocortin (POMC), and cocaine- and amphetamine-regulated transcript (CART) neurons, which activate anorexigenic pathways [67, 68] and agouti-related peptide (AGRP) and neuropeptide Y (NPY) neurons that transfer appetite stimulating signals. A decrease in leptin is correlated with an increased food intake [67]. 

By binding to LEPR in the hypothalamus, leptin causes Janus kinase 2 (JAK2) activation and LEPR tyrosine residues phosphorylation, allowing STAT3 to be dimerized and translocated to the nucleus, leading to anorectic peptide synthesis [67, 68]. Also, it has been shown that leptin's effects on food intake and body weight require inhibition of hypothalamic AMPK. Thus, hypothalamic AMPK plays a critical role in hormonal and nutrient-derived anorexigenic and orexigenic signals and in energy balance [69, 70]. 

### 3.3. Plasminogen Activator Inhibitor-1 (PAI-1)

Plasminogen activator inhibitor-1 (PAI-1) is the most important endogenous inhibitor of fibrinolysis and increased levels are associated with insulin resistance, body weight control, and thrombosis. In humans, visceral adipose mass has been shown to be a primary determinant of PAI-1 levels. In T2DM, not only increased adipose tissue mass but other metabolic disturbances, including hyperinsulinemia, hyperglycemia, and dyslipidemia, alter adipose tissue function and lead to increased production and circulating levels of PAI-1 [25]. Consumption of fructose at 25% of energy requirements for 10 weeks leads to increases of fasting as well as postprandial PAI-1, suggesting the possibility that prolonged consumption of fructose may contribute to the development of metabolic syndrome via induction of prothrombotic (PAI-1) mediators besides proinflammatory cytokines [71].

## 4. Combined Signals

**Figure 2:**
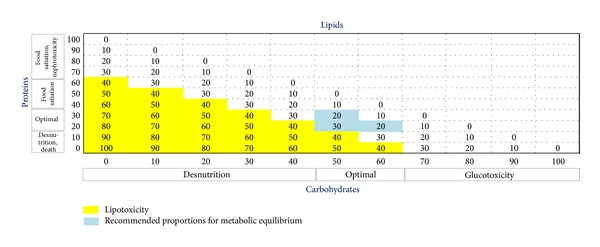
Recommended proportions of macronutrients.

In the fed physiological state a multitude of gut hormones are released into the circulation at the same time depending on the quality and quantity of the diet with recommended proportions of the macronutrients as follows: carbohydrates 60%, proteins: 20%, and lipids: 20% (Figure 2). How the satiety factors act in concert to regulate appetite is still misunderstood. 

Following the above-mentioned idea, after a high-protein meal, ghrelin declines gradually in both normal weight and obese children without subsequent increase, whereas ghrelin is suppressed more rapidly to a nadir at 60 min after a high-carbohydrate meal in both groups of children, followed by rebound in ghrelin levels. Similarly, after the high-protein meal, PYY concentrations increase steadily over the course of the morning in both groups without decline, whereas PYY levels peaked 30 min after the high-carbohydrate meal in both normal weight and obese subjects with significant decline thereafter. Ghrelin and PYY responses to the high-fat meal are somewhat intermediate between that observed with high carbohydrate and high protein [15].

Amylin, especially when combined with other anorectic hormones, has beneficial long-term effects on body weight. For example, amylin and GLP-1 mediate the feedback control of eating by seemingly separate but overlapping mechanisms. Another case is CCK, a synergic effect has been observed when applied simultaneously with amylin, estradiol, insulin, and leptin [72].

The combination of PYY_3-36_ and GLP-17-36 amide produces a reduction in *ad libitum* energy intake in healthy, lean human subjects [73]. Recent work in investigating the utility of combinational therapies for the treatment of obesity has focused on the coadministration of amylin with leptin [74]. Moreover, combinational therapy of exendine-4 + sCT produced sustained daily food reductions without tolerance, nausea, malaise, or rebound feeding. These findings further support the view that engaging multiple feeding inhibitory pathways to reduce food intake could be a potential strategy for the treatment of obesity.

## 5. Peripheral Signals Modulated by Food

One strategy for the prevention of overweight and obesity related diseases is the use of agents that interfere with the hydrolysis and absorption of dietary carbohydrates and lipids. The most important dietary carbohydrates are starch, sucrose, and lactose. They are digested by disaccharidases in the upper gastrointestinal tract and broken down into monosaccharides. Subsequently they are absorbed to the circulation. The elevated glucose concentration in blood promotes insulin secretion from the *β* cells of the islets of Langerhans in the pancreas, and insulin mediates the uptake of glucose in peripheral tissues including muscle, adipose tissue, and kidney. Taking into account the importance of carbohydrate metabolism, the gastrointestinal enzymes can be therapeutic targets for limiting absorption of monosaccharides [75]. 

In addition, the most important dietary lipids are triglycerides and cholesterol esters. They are digested by pancreatic lipase and pancreatic phospholipase A2 to glycerol, fatty acids, and free cholesterol. Finally, they are absorbed to the circulation and may be used or stored in adipose tissue [76].

In the literature it can be found several reviews that describe active substances in plants that inhibit pancreatic enzymes. It has been recorded that more than 1200 plant species could have a hypoglycemic activity [77]. For example, Hanhineva et al. revised the inhibitory properties of polyphenols (i.e. flavonoids, phenolic acids, proanthocyanidins, and resveratrol). They reported that these polyphenols may influence carbohydrate metabolism at many levels. More interesting is that these compounds are contained in plant-based foods, such as tea, coffee, wine, cocoa, cereal grains, soy, fruits, and berries [78, 79]. 

Besides the food in their natural form, the heat processing of food (i.e., boiling) can produce derivate compounds that show digestive enzymes inhibitory properties. For example, it has been shown that after heat treating of raw ginseng, amino acid derivatives such as arginyl-fructose and arginyl-fructosyl-glucose are formed at high levels; these products inhibited postprandial hyperglycemia through the inhibition of *α*-amylase and *α*-glucosidase [80]. Other compounds such as flavonoids from grape seed Cat's whiskers and Sweetleaf extract obtained by heat processing also inhibited *α*-amylase [81].

It is worth mentioning that some plants do not show a significant effect in the inhibition of *α*-amylase; however, in combination with acarbose, an antidiabetic drug with *α*-glucosidase inhibitory properties has a synergistic effect due to low doses of acarbose that are necessary for the postprandial glycemic control. For example, the polyphenol extracts from a range of berries, especially raspberry and rowanberry, showed an effect only in combination with acarbose [82]. Other examples of this synergistic action are the inhibition by cyanidin-3-rutinoside [83] and some species of cinnamon [84]. 

The brush border enzymes are inhibited by molecules extracted from plants. For example, the D-fagomine from seeds of buckwheat inhibits sucrase [85], diacylated anthocyanin from purple sweet potatoes has been shown to inhibit maltase [86], cyanidin-3-galactoside inhibits sucrase and maltase [87], and *α*-glucosidase is inhibited by the hydro-methanolic seed extract of *Holarrhena antidysenterica* [88], by ethanol extract of the fruit case of *Garcinia mangostan* [89], and by *Corni fructus* [90]. Flavonoids from grape seed Cat's whiskers and Sweetleaf extract inhibit intestinal sucrase and maltase [81] (which is also inhibited by some species of cinnamon) [84].

Enzymes from the metabolism of lipids can be also inhibited by compounds of the plants; for example, oligomeric procyanidins in the apple polyphenol extract inhibit pancreatic lipase [91], and arginyl-fructose and arginyl-fructosyl-glucose inhibit lipase [80]. Cocoa procyanidins inhibit pancreatic lipase, also pancreatic *α*-amylase, and phospholipase A2, and this inhibition produces a decrement in plasma triglyceride and glucose concentrations in mice as well as humans [92].

More studies are needed about the inhibitory activity of substances from natural origins (i.e., plants) on intestinal enzymes. Diabetic patients would beneficiate if they include these plants in their diet instead of active purified compounds. However, the concentrations in food of the active complexes could be not enough, then it is essential to get them in a purify form. For this reason, studies are needed in this area.

## 6. Evidence for the Existence of an Intestinal Missing Link

### 6.1. Gestational Diabetes Mellitus

Obesity increases the risks of gestational diabetes mellitus (GDM) [93, 94]. Even more, there seems to be a shared genetic basis between GDM and T2DM [95]. In fact, the diagnosis of GDM identifies patients with a pancreatic *β*-cell defect. In some patients, the defect is transient or stable, but in most it is progressive, imparting a high risk of diabetes for at least a decade after the index pregnancy. 

The majority of women with GDM have clinical characteristics indicating a risk for T2DM. Available evidence indicates that T2DM can be prevented or delayed by intensive lifestyle modification and by medications, particularly those that ameliorate insulin resistance. All patients should be monitored for rising glycemia indicative of progressive *β*-cell deterioration. Monitoring should be initiated at least annually and should be intensified if glycemia is rising and/or impaired. 

Like monitoring, lifestyle modification for obese and overweight women during pregnancy should be intensified for rising glycemia and/or development of impaired glucose levels. These measures restrict gestational weight gain and reduce the prevalence of gestational diabetes [96].

### 6.2. Obesity and Diabetes

Obesity, a BMI greater than 30 kg/m^2^, is strongly and causally linked to T2DM. Recent data suggest that the prevention of diabetes is feasible if weight management is addressed. Modest weight loss of 5–10% body weight is known to improve diabetes by reducing insulin resistance in obese individuals [97]. Regarding this strategy, in clinical trials, caloric restriction, exercise, and weight loss have been shown to prevent and reduce diabetes in obese individuals [97, 98] in part by attenuating insulin resistance and subsequent hyperinsulinemia, thereby preserving *β*-cell function [99, 100].

While the goal of a cure for T2DM remains some way off, bariatric surgery has long been proven to be effective in weight reduction in the morbidly obese, as well as in maintaining long-term weight reduction. With this weight reduction, obesity-related comorbidities, including T2DM, tend to improve or resolve completely. 

### 6.3. Bariatric Surgery

Bariatric surgery promotes effective and sustained weight loss in morbidly obese subjects [101]. Since 1991, several medical societies have established the criteria for bariatric surgery in cases with BMI > 40 or BMI > 35 with serious comorbidities [102]. 

Depending on the type of bariatric procedure, up to 80% resolution of T2DM has been reported, being more effective that those techniques that bypass the foregut like the Roux en-Y gastric bypass (RYGBP) [103–106]. Being more specific, Sugerman et al. found that a young age was a positive predictor for T2DM resolution [107] as well as early surgery that preempts irreversible pancreatic *β*-cell deterioration [108].

There are two different theories proposed to explain the laboratorial benefits after bariatric surgery. The hindgut theory by Cummings et al. [109] proposes that the rapid transit of nutrients to the hindgut improves glucose metabolism, probably through GLP-1. The second hypothesis, the foregut theory by Rubino [110], says that the exclusion of the foregut from the food stream causes a decrease in insulin resistance through the secretin pathway. The two theories are not mutually exclusive.

RYGBP causes an improvement in a diabetic patient's status through a variety of mechanisms. More interestingly, improvement often occurs very soon after the bypass, even before significant weight loss has occurred [111, 112]. First and foremost, RYGBP enforces severe calorie restriction through both mechanical restriction and the upregulation of satiety signals such as anorexigen PYY [113]. The decrease in caloric intake is by itself able to result in the improvement of T2DM [114]. 

One possible explanation for the metabolic improvement after RYGBP hypothesis is that bariatric surgeries with gastric bypass exclude the site responsible for the production of the hormone causing T2DM [115]. Other explanations are possible. For example, a hormone overproduced in the proximal foregut in diabetic patients might counteract the action of insulin, thus inducing insulin resistance and only secondarily hyperinsulinemia. 

Collectively, evidence supports the concept that the effect of bariatric surgery on diabetes is mediated by a change in the pattern of secretion of gastrointestinal hormones [116], supporting the use of them as therapeutic targets [117–119]. As a first instance, there is a greater insulin sensitivity due to a better *β*-cell function including the first phase of insulin secretion [120]. Also, there is restoration of a near-normal, postprandial insulin response soon after RYGBP [121], which is associated with a rise in GLP-1 levels [122]. Even more, ghrelin levels fall after RYGBP [109], resulting in appetite reduction. 

It has been proposed that the bypass of the foregut in RYGBP restores normal GIP sensitivity and normalises the GIP levels [111], breaking the “GIP-resistant state,” present in T2DM [123].

With the strong evidence published worldwide, surgery has been proven to be superior to medical treatment in terms of maintaining weight loss and altering the natural course of T2DM, which has been considered medically incurable [108, 124]. Despite the obvious risks of surgery [125], the risks of morbid obesity as well as all its associated comorbidities make surgery a viable option in those who are eligible.

## 7. The Adipose-Intestinal Missing Link

In susceptible individuals, chronic exaggerated stimulation of the proximal gut with fat and carbohydrates may induce overproduction of an unknown factor that causes impairment of incretin production and/or action, leading to insufficient or untimely production of insulin, so that glucose intolerance develops. 

The bypass of the duodenum and jejunum might avoid a putative hormone overproduction in the proximal foregut in diabetic patients that might counteract the action of insulin, while the early presentation of undigested or incompletely digested food to the ileum may anticipate the production of hormones such as GLP1, further improving insulin action [126]. Moreover, GLP-1 has been implicated in the differentiation of pancreatic exocrine cells toward *β* cells by the Pdx-1 gene transcription stimulation. Indeed, GLP-1 increases the expression of *β*-cell-specific genes such as insulin, glucose transporter 2 (GLUT2), and glucokinase in human and rat pancreatic ductal cells transfected with Pdx-1 compared with those transfected with null vector [127].

Carbohydrates are mostly digested to glucose, fructose, and galactose before absorption by the small intestine. Absorption across the brush border and basolateral membranes of enterocytes is mediated by Na^+^-dependent and -independent membrane proteins. Glucose and galactose transport across the brush border occur by a Na^+^/glucose (galactose) cotransporter (SGLT1), whereas passive fructose transport is mediated by a uniporter (GLUT5). The passive exit of all three sugars out of the cell across the basolateral membrane occurs through two uniporters (GLUT2 and GLUT5). Mutations in SGLT1 cause a major defect in glucose and galactose absorption (glucose-galactose malabsorption), but mutations in GLUT2 do not appear to disrupt glucose and galactose absorption [128].

Because bariatric surgery with bypass obviates a great area of disaccharidases action, it is expected a reduction in glucose absorption which consequently leads to hyperglycemia improvement. Notwithstanding, a metabolic control would not be registered if there was not a *β*-cell recovery. 

The common variable in the pathogenesis of GDM and T2DM is the weight gain surpassing recommended BMI. Furthermore, keeping a normal weight is fundamental in the prevention of these pathologies that are cured after a great weight loss coming in the puerperium or with bariatric surgery, respectively. This implies the role of circulating adipose signal acting on the proximal intestine that might inactivate a critical factor for the metabolic homeostasis (mainly insulin effect). SGLT1 or disaccharidases might be two target candidates to be affected by this adipose tissue derived factor.

Tumour necrosis factor *α* (TNF-*α*) expressed in high circulating levels in obesity is a proinflammatory cytokine implicated in the induction of insulin resistance [129]. There is also evidence of TNF-*α* effects on the *β* cell, which may further contribute to T2DM, although, as this cytokine is expressed in many other diseases without causing T2DM is low the probability to be by itself the adipose-intestinal link of T2DM. 

## 8. Conclusions

The combined effects of macronutrients on the predominant gut hormone secretion are still poorly understood. Thus, from a therapeutic perspective, targeting the interaction of appetite signals in the gut offers the potential advantage of being able to manipulate appetite at a site distant from the CNS through endocrine and vagal nerve mechanisms [130].

Finally, future studies will target the identification of a proximal intestinal metabolic molecule, implicated as the cause or cure of T2DM whether activated or not. 

## Figures and Tables

**Table 1:** Major peripheral signals involved in food intake regulation.

	Hormone	Site of secretion	Major receptors	Major actions
Intestinal	Amylin	Pancreatic *β* cells	AMY_1–3_	Inhibits gastric secretion. Delays gastric emptying. Decreases blood glucose.
	Cholecystokinin	Intestinal Icells	CCK2	Gall bladder contraction. Delays gastric emptying. Pancreatic enzyme contraction.
	Endocannabinoid system	Postsynaptic cell	CB1, CB2	Modulates appetite besides a variety of physiological processes.
	Ghrelin	Gastric fundal A cells	GHS–R	Increases gastric motility. Growth of hormone release.
	Glucagon	Pancreatic *α* cells	Glucagon	Gluconeogenesis. Glycogenolysis.
	Glucagon-like peptide-1 (GLP-1)	Gastrointestinal L cells	GLP–1	Glucose-dependent insulin release. Delays gastric emptying. Vagal and CNS effects.
	Glucose-dependent insulinotropic polypeptide (GIP)	K cells in duodenum and jejunum	GIP–R	Stimulates insulin synthesis and secretion.
	Oxyntomodulin	Gastrointestinal L cells	GLP–1	Glucose-dependent insulin release. Delays gastric emptying. Vagal and CNS effects.
	Pancreatic polypeptide	Pancreatic PP cells	Y_4_	Delays gastric emptying.
	Peptide YY (PYY)	Gastrointestinal L cells	Y_2_	Delays gastric emptying. Vagal and CNS effects.
Adipose	Adiponectin	Adipocyte, skeletal muscle, endothelial cells, and cardiomyocytes	AdipoR1 AdipoR2 T-cadherin	Adiponectin, via AMPK phosphorylation, increases insulin sensitivity, fatty acid oxidation and reduces the synthesis of glucose in the liver and other tissues.
	Leptin	Adipocyte	LEPR	Increases POMC anorexigenic signals. Inhibits NPY, stimulating appetite.
	Plasminogen activator inhibitor-1 (PAI-1)	Endothelium, adipocyte	Binds to (tPA)	Inhibitor of fibrinolysis.
	Tumour necrosis factor alpha (TNF-*α*)	Adipocyte	Tumor necrosis factor receptor (TNFR)	Insulin resistance.

AMPK: AMP-activated protein kinase, CNS: central nervous system, NPY: neuropeptide Y, POMC: proopiomelanocortin, and tPA: tissue plasminogen activator.

**Table 2:** BMI, age, sex, and gut hormones.

	Children		Female									Male			Adults		
Age	9.62 ± 0.42 [15]	9.68 ± 0.29 [15]	24 ± 8 [16]	24 ± 5 [16]	15 ± 1 [17]	41.2 ± 12.9 [18]	53.5 ± 10.8 [19]	14 ± 1 [17]	47.9 ± 7.8 [20]	47.9 ± 7.8 [20]	46.6 ± 13.1 [21]		53.5 ± 10.8 [19]	51 ± 7 [22]	53.5 ± 10.8 [19]	47.1 ± 2.5 [23]	45.0 ± 2 [23]
BMI (kg/m^2^)	17.3 ± 0.4 [15]	27.9 ± 1.1 [15]	15.4 ± 1.4 [16]	20.9 ± 1.9 [16] 21.1 ± 0.6 [24]	22.2 ± 0.7 [17]	25.4 ± 4.9 [18]	30.3 ± 6.1 [19]	33.0 ± 3.3 [17]	39.0 ± 3.8 [20]	42.8 ± 3.8 [20]	47.1 ± 88.1 [21]		30.3 ± 6.1 [19]	42.1 ± 7.0 [22]	30.3 ± 6.1 [19]	41 ± 1 [23]	48 ± 1 [23]
CCK (pmol/L)					1.4 ± 0.1 [17]			0.5 ± 0.2 [17]									
Leptin (*µ*g/L)	3.52 ± 0.85 [15]	31.8 ± 4.0 [25]	2.3 ± 1.3 [16]	12.0 ± 6.73 [16]		18.5 ± 11.6 [18]	26.8 [19]		22.0 ± 12.3 [20]	30.6 ± 11.7 [20]						36.8 ± 2.7 [23]	38.2 ± 2.0 [23]
Ghrelin (pg/mL)	835 ± 47 [15]	664 ± 37 [15]					881.7 ± 413.5 [19]		519 ± 105 [20]	425 ± 91 [20]			764.5 ± 292.5 [19]	632 ± 99 (pmol/L) [22]	817.0 ± 355.5 [19]	263 ± 34 [23]	330 ± 25 [23]
GIP (pmol/L)					37 ± 7 [17]			41 ± 5 [17]									
GLP-1 (pmol/L)									6.12 ± 4.19 [20]	6.02 ± 2.77 [20]	0.65 ± 0.18 (ng/mL) [21]	5 − 10 [26]				8.2 ± 1.5 [23]	5.8 ± 1.4 [23]
PYY (pg/mL)	87.0 ± 7.9 [15]	111.1 ± 11.0 [15]		86 (pg/L) [24]					69.5 ± 41.3 [20]	69.4 ± 44.3 [20]						130 ± 17 [23]	90 ± 19 [23]

BMI: body mass index, CCK: cholecystokinin, GIP: gastric inhibitory peptide, GLP-1: glucagon-like peptide-1, and PYY: peptide YY.
